# Slr1670 from *Synechocystis* sp. PCC 6803 Is Required for the Re-assimilation of the Osmolyte Glucosylglycerol

**DOI:** 10.3389/fmicb.2016.01350

**Published:** 2016-08-29

**Authors:** Philipp Savakis, Xiaoming Tan, Cuncun Qiao, Kuo Song, Xuefeng Lu, Klaas J. Hellingwerf, Filipe Branco dos Santos

**Affiliations:** ^1^Molecular Microbial Physiology Group, Swammerdam Institute for Life Sciences, University of AmsterdamAmsterdam, Netherlands; ^2^Key Laboratory of Biofuels, Shandong Provincial Key Laboratory of Energy Genetics, Qingdao Institute of Bioenergy and Bioprocess Technology – Chinese Academy of SciencesQingdao, China

**Keywords:** Slr1670, cyanobacteria, *Synechocystis*, osmolyte, glucosylglycerol, salt stress

## Abstract

When subjected to mild salt stress, the cyanobacterium *Synechocystis* sp. PCC 6803 produces small amounts of glycerol through an as of yet unidentified pathway. Here, we show that this glycerol is a degradation product of the main osmolyte of this organism, glucosylglycerol (GG). Inactivation of *ggpS*, encoding the first step of GG-synthesis, abolished *de novo* synthesis of glycerol, while the ability to hydrolyze exogenously supplied glucoslylglycerol was unimpaired. Inactivation of *glpK*, encoding glycerol kinase, had no effect on glycerol synthesis. Inactivation of *slr1670*, encoding a GHL5-type putative glycoside hydrolase, abolished *de novo* synthesis of glycerol, as well as hydrolysis of GG, and led to increased intracellular concentrations of this osmolyte. Slr1670 therefore presumably displays GG hydrolase activity. A gene homologous to the one encoded by *slr1670* occurs in a wide range of cyanobacteria, proteobacteria, and archaea. In cyanobacteria, it co-occurs with genes involved in GG-synthesis.

## Introduction

Upon increases in extracellular osmolarity, many bacteria synthesize small organic molecules that raise the intracellular osmotic pressure. In cyanobacteria, the nature of the osmolyte correlates with the host’s osmotolerance: strains with a low salt tolerance produce sucrose; moderately halotolerant strains utilize glucosylglycerol (GG); and highly tolerant strains use glycine betaine (Hagemann, 2011).

**FIGURE 1:**
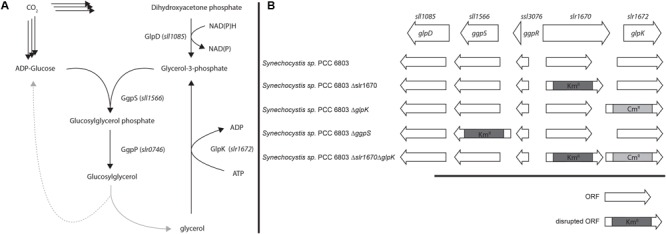
**(A)** Proposed overview of Glycerol and glucosylglycerol (GG) metabolism. **(B)** Genes involved in GG synthesis and mutant strains used in this study. Arrows represent the direction of transcription of the respective open reading frame.

The moderately halotolerant cyanobacterium *Synechocystis* sp. PCC 6803 (hereafter: *Synechocystis*) uses GG as its primary osmolyte (Richardson et al., 1983), but is also capable of salt-induced sucrose synthesis. GG is synthesized via a two-step pathway from central metabolites (**Figure 1**): in the first step, glucosylglycerol phosphate is synthesized from ADP-glucose and glycerol-3-phosphate, in a condensation reaction catalyzed by glucosylglycerol phosphate synthase (GgpS). Cleavage of the phosphate moiety is subsequently accomplished by glucosylglycerol phosphate phosphatase (GgpP). *Synechocystis* harbors a transporter that is used for reuptake of GG that is lost due to leakage of this osmolyte from the cytoplasm (Hagemann et al., 1997).

Another osmolyte that is frequently used by bacteria such as *Escherichia coli* is the disaccharide trehalose (Welsh et al., 1991). Although incapable of its synthesis, *Synechocystis* can take up exogenously supplied trehalose and use it as an osmoprotectant (Mikkat et al., 1997). In agreement with this it is observed that in cells to which trehalose has been added, the total concentration of GG decreases over time, suggesting that this latter molecule can be catabolized (Mikkat et al., 1997).

Some marine cyanobacteria have been demonstrated to be able to ferment a part of their osmolytes. Thus, *Microcoleus chthonoplastes* ferments some of its GG in the dark, re-assimilating the glucose part, while the glycerol part is excreted, or lost through leakage through the cytoplasmic membrane (Stal and Moezelaar, 1997). Yet, a metabolic pathway for GG re-assimilation has so far remained elusive in cyanobacteria (Pade and Hagemann, 2014), although recently, a dedicated GG phosphorylase was discovered in *Bacillus selenitireducens* (Nihira et al., 2014).

We reported elsewhere that under mild salt stress, wild-type *Synechocystis* cells synthesize small quantities of glycerol (Savakis et al., 2015). Here, we provide evidence supporting the notion that GG is the source of this glycerol under salt stress. In addition, we demonstrate that a previously unassigned protein, Slr1670, is directly involved in (and required for) GG re-assimilation.

## Materials and Methods

Chemicals were purchased from Sigma-Aldrich, unless stated otherwise. Glucosylglycerol (GG, 51% aqueous solution) was purchased from Bitop (Germany).

### Culturing Conditions

*Escherichia coli* was grown in LB medium at 37°C and shaking at 200 rpm. Selection of transformants was carried out at 37°C on LB medium solidified with 1.5% (w/v) agar. Where appropriate, ampicillin, kanamycin and chloramphenicol were added at 100, 50, and 35 μg/mL, respectively.

For batch experiments, *Synechocystis* cells were grown in a shaking incubator (Innova 43, New Brunswick Scientific, 120 rpm, 30°C) under fluorescent white light (15 W cool fluorescent white light, F15T8-PL/AQ, General electric, incident light intensity 30–40 μE/m^2^/s) in BG11 medium, supplemented with 10 mM TES/KOH and adjusted to an initial pH of 8.0. Cells were inoculated to an OD_730_ of 0.1 from a pre-culture. Where indicated, NaCl was added to a concentration of 200 mM to non-adapted cells.

For salt tolerance experiments, cells were grown in transparent 96-well plates [Greiner bio-one cellstar, F-bottom with breathe easy seals (Diversified Biotech)] under white light (GE PL/AQ F15T8) at 30°C and shaking at 700 rpm in BG11 medium, buffered to an initial pH = 8.0 with 10 mM TES/KOH and supplemented with NaHCO_3_ to a concentration of 50 mM.

*Synechocystis* mutants were selected under white light (10 μE/m^2^/s) at 30°C on BG11 medium solidified with 1.5% (w/v) agar and supplemented with 10 mM TES/KOH pH = 8.0, plus 0.3% (w/v) Na_2_S_2_O_3_ and selection markers where appropriate.

### Extraction of Genomic DNA

Cells were grown until an OD_730_ of around 1 and 1 mL was harvested by centrifugation [12,000 rpm, 1 min, room temperature (RT)]. The supernatant was discarded and the cells were resuspended in 200 μL TE buffer (10 mM Tris-HCl, 1 mM EDTA, pH 8.0). Next, 200 mg glass beads (0.1 mm diameter) were added and the sample was vortexed for 5 min. The sample was cleared by centrifugation (5 min, 12,000 rpm, RT), and the supernatant was transferred to a fresh tube. Then, Phenol/chloroform/isoamylalcohol (25/24/1, v/v/v; 200 μL) was added, the sample was mixed and centrifuged (5 min, 12,000 rpm, RT). The aqueous phase was transferred to a fresh tube and washed with 200 μL of chloroform/isoamylalcohol (24/1, v/v). After centrifugation (5 min, 12,000 rpm, RT), 40 μL 5 M NaCl and 400 μL ethanol were added. After thorough mixing, the sample was placed at -20°C for 30 min. Then, the DNA was pelleted (10 min, 12,000 rpm, RT) and washed with 500 μL 70% pre-cooled ethanol. After centrifugation (5 min, 12,000 rpm, RT), the supernatant was removed and dried on a bench-top incubator at 30°C for 15 min. The pellet was dissolved in 30 μL nuclease-free water and used immediately, or stored at -20°C.

### Plasmid Construction

For an overview of the plasmids used and created in this study, see **Table 1**. For the primers, see **Table 2**. For the construction of pMD18Tslr1670, *slr1670* was amplified from genomic DNA of *Synechocystis* with primers slr1670-BamHI and slr1670-XhoI and introduced into pMD18-T. For the construction of pMD18Tslr1670KmR, pMD18Tslr1670 was digested with NcoI, blunted with T4 DNA polymerase and ligated with the Km resistance cassette (obtained from pRL446 Elhai and Wolk, 1988 by digestion with PvuII). The *glpK* gene was amplified from the genomic DNA of *Synechocystis* by PCR with primers glpK-Fwd and glpK-Rev, and cloned into the pMD 18-T vector (Takara, Japan). The resulting plasmid was digested by NheI, blunted by T4 DNA Polymerase (Fermentas), and ligated to the blunted chloramphenicol resistance gene cassette, resulting in the plasmid pXT323.

**Table 1:** Plasmids used in this study.

Plasmid	Description	Source
pRL446		Elhai and Wolk, 1988
pMD18T	TA cloning vector	TaKaRa clonetech
pMD18Tslr1670		This study
pMD18Tslr1670KmR	Construction Δ*slr1670*	This study
pXT323	Construction Δ*glpK*	This study

**Table 2:** Primers used in this study.

Primer	Sequence
Slr1670-BamHI	AGGATCCATGAAAACATTGAATCGTATCCATCTG
Slr1670-XhoI	TCTCGAGGCATTCCTTCTTCGAGCGA
Slr1670-NheI fw	GATAACGCTAGCATGAAAACATTGAATC
Slr1670-BamHI rv	ATGGATCCCTAGCATTCCTTCTTCGAGC
glpK-Fwd	CGCCATATGACAGCAAAACATAATCAG
glpK-Rev	TCTCGAGGATGGAAGCAATGTCAC

### Construction of Mutant *Synechocystis* Strains

For an overview of the strains in this study, see **Table 3**. Mutant strains of *Synechocystis* were constructed essentially as described previously (Vermaas, 1996; Angermayr et al., 2012). Briefly, 10 mL cells were grown to an OD_730_ of 0.2–0.6, centrifuged and concentrated 20- to 50-fold. Then, plasmid DNA (1–3 μg) was added to 100–300 μL of the cell suspension. The cells were incubated in tubes at 30°C for 5 h. Cells were grown for 16 h on a membrane filter, on plates without selective pressure. Next, cells were transferred onto plates containing the relevant selection marker. Colonies appeared after 1–2 weeks. Identity of transformants was confirmed by colony PCR, using appropriate primers. Segregation of mutant strains was achieved in liquid culture. For construction of the *slr1670* strain, WT *Synechocystis* was transformed with pMD18Tslr1670KmR. For construction of the double insertion mutant Δ*slr1670*Δ*slr1672, Synechocystis* Δ*slr1672* was transformed with pMD18Tslr1670KmR, and segregation was monitored by PCR (with the primers NheI-slr1670 fw and BamHI-slr1670 rv).

**Table 3:** Strains used in this study.

Strain	Genotype	Source
*Synechocystis* sp. PCC 6803		X. Xu, Institute of Hydrobiology – Chinese Academy of Sciences
*Synechocystis ΔggpS*	*ΔggpS*::Km^R^	Du et al., 2013
*Synechocystis ΔglpK*	*ΔglpK*::Cm^R^	This study
*Synechocystis Δslr1670*	*Δslr1670*::Km^R^	This study
*Synechocystis ΔglpKΔslr1670*	*ΔglpK*::Cm^R^*Δslr1670*::Km^R^	This study

### Analysis of Intra- and Extracellular Metabolites Using HPLC

For analysis of extracellular metabolites, samples from a culture were cleared by centrifugation (14,500 rpm, 10 min, 21°C) and remaining particles were removed with a syringe, fitted with a filter (Sartorius Stedin Biotech, minisart SRP 4, 0.45 μm pore size). Samples were then analyzed by HPLC [column: Rezex ROA-Organic Acid H^+^ (8%) (Phenomenex); column temperature: 85°C; detector (Jasco, RI-1530); eluent: 7.2 mM H_2_SO_4_; flow: 0.5 mL/min]. Identification and quantification was done using external standards (detection limit: ∼0.02 mmol/L).

For extraction of GG from cyanobacteria, 1 mL of a culture (OD_730_ = 6) was harvested by centrifugation (15,000 rpm, 10 min, 4°C). Pellets were resuspended in 1 mL 80% (v/v) ethanol and incubated at 65°C for 4.5 h. After re-centrifugation (15,000 rpm, 10 min, 21°C), the supernatant was transferred to a fresh tube and the liquid was evaporated using a stream of nitrogen gas. The residue was dissolved in 1 mL of water, filtered and analyzed by HPLC as described above.

### Genome Scale Metabolic Modeling

**FIGURE 2:**
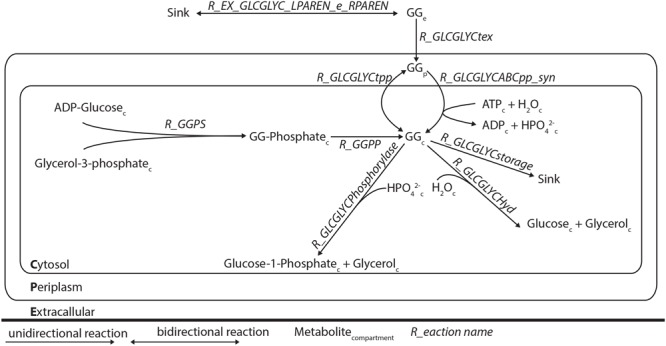
**Schematic representation of the modifications of the genome-scale model of *Synechocystis* sp. PCC 6803**. The reactions R EX GLCGLYC LPAREN e RPAREN, R GLCGLYCtex, R GLCGLYCtpp, R GLCGLYCABCpp syn, R GLCGLYCstorage, R GLCGLYCHyd, and R GLCGLYCphosophorylase were added.

We studied the newly identified GG degradation pathway in the context of a genome-scale metabolic network model previously reported for *Synechocystis* (Nogales et al., 2012). Although GG was already present in this earlier reconstruction, along with the reactions needed for its synthesis and exchange over the cytoplasmic membrane, some missing key steps made it impossible for the degradation pathway to carry any flux *in silico*. In order to fix this, and additionally include the re-utilization of GG, reactions for transport, metabolism and storage were added to the model (**Table 4**; **Figure 2**). Flux balance analyses of this new version of the *Synechocystis* stoichiometric model were carried out in the on-line modeling platform FAME (Boele et al., 2012), using newly developed visualization tools specific for this organism (Maarleveld et al., 2014). Constraining the relevant reactions correctly simulated the phenotype of the different derivative strains constructed in this study. The stoichiometric impact of the different GG breakdown pathways that are postulated here on the genome-scale metabolic network of *Synechocystis* was assessed by including the two alternative routes in the model. Comparisons between model versions mimicking different strains and conditions were established using biomass maximization as the objective function, while constraining the exchange flux of GG according to experimental measurements, as detailed elsewhere (Santos et al., 2011). Increased fitness was deduced from calculations of the maximum of the objective function (BOFmax) for the unconstrained utilization reaction (i.e., GG phosphorolysis, hydrolysis, and/or glycerol phosphorylation constrained between 0 and ∞), divided by BOFmax for the respective flux constrained to zero.

**Table 4:** Reactions added to the genome-scale model of *Synechocystis.*

Reaction name	Reaction ID	Equation	Gene association
GG transport via diffusion (cytosol to periplasm)	R_glcglyctpp	M_glcglyc_c⇒M_glcglyc_p	–
Glucosylglycerol Hydrolase	R_GLCGLYCHyd	M_h2o_c + M_glcglyc_c⇒M_glc_DASH_D_c + M_glyc_c	*slr1670*
Glucosylglycerol Phosphorylase	R_GLCGLYCPhosphorylase	M_pi_c + M_glcglyc_c⇒M_g1p_c + M_glyc_c	*slr1670*
Glucosylglycerol storage (cytosol to sink)	R_GLCGLYCstorage	M_glcglyc_c⇒	–

*M_glcglyc, glycosylglycerol; M_h2o_c, water; M_glc_DASH_D, D-glucose; M_glyc, glycerol; M_pi, inorganic phosphate; M_g1p, glucose-1-phosphate.*

### Phylogenetic Analyses

**FIGURE 3:**
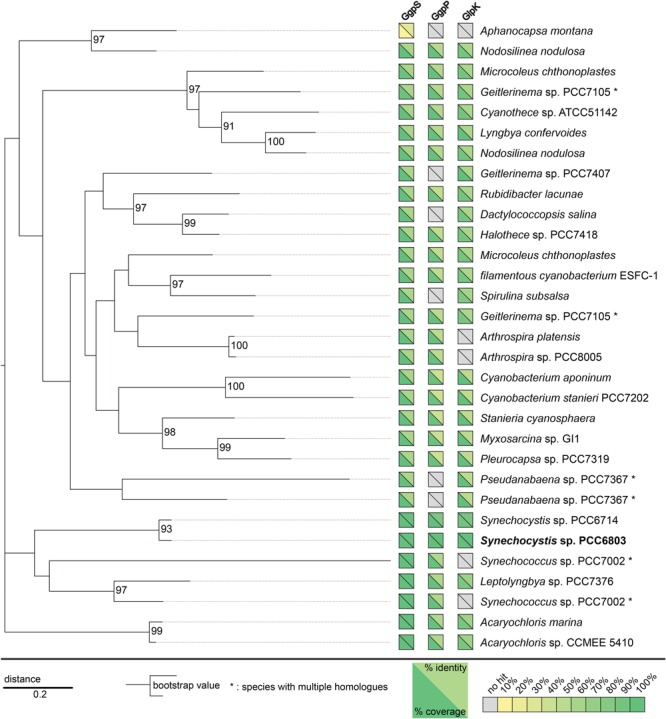
**Slr1670 homologs are present in a variety of cyanobacterial-, bacterial-, and archaeal species.** A phylogenetic tree based on the similarity of these homologs is shown for cyanobacterial species for which a complete genome sequence was available (see supplement for the full tree). Additionally, yellow-green boxes visualize presence of genes homologous to *ggpS, ggpP*, and *glpK*, in the respective genome. Gray boxes indicate that no significant hit was obtained. Yellow triangles mean low coverage/similarity while green triangles indicate high coverage/similarity.

PSI-BLAST of the Slr1670 sequence against non-redundant protein sequences was carried out on the 24th of September, 2015, using the following parameters: expect threshold: 10, word size: 3, matrix: BLOSUM62, Gap existence cost: 11, Gap extension cost: 1, Conditional compositional score matrix adjustment. For the second iteration, sequences with a coverage > 90% (91/96 hits) were selected. Alignments were constructed using MEGA6 (ClustalW algorithm; pairwise alignment: gap opening penalty: 6, gap extension penalty: 0.1; Multiple alignment: gap opening penalty: 6, gap extension penalty: 0.2; Protein weight matrix: BLOSUM62; Residue-specific penalties: on; Hydrophilic penalties: on; Gap separation distance: 4; end gap separation: off; use negative matrix: off; delay divergent cutoff: 30%). The best model for the phylogenetic analysis was found using the following parameters: tree to use: neighbor-joining tree; statistical method: maximum likelihood; Gaps/missing data treatment: partial deletion; site coverage cut-off: 95%; branch swap filter: very strong. The best model for the cyanobacterial subset (**Figure 3**) was LG+G (Le and Gascuel, 2008). The best model for the entire set of proteins was LG+G+F (Le and Gascuel, 2008). The outgroup (hexokinase 1 of *Saccharomyces cerevisiae*) was used to root the trees and was not included in the figures.

## Results

**FIGURE 4:**
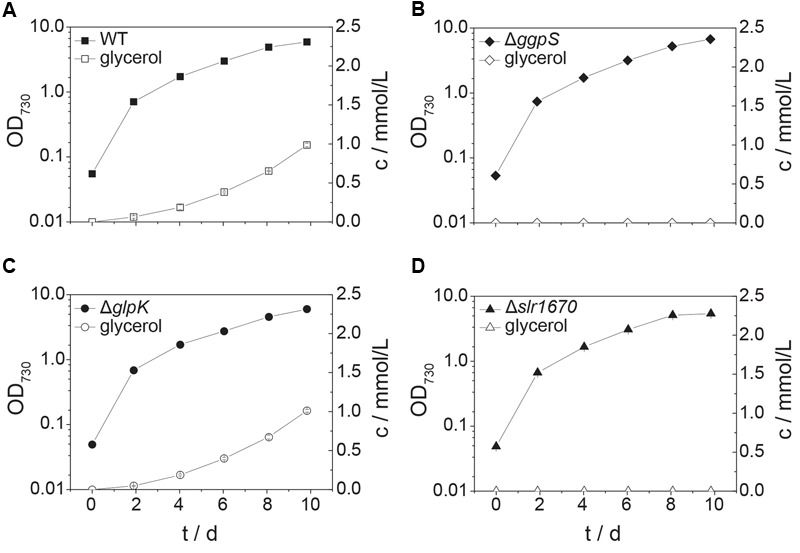
**Inactivation of *slr1670* or *sll1566* (*ggpS*) abolishes glycerol production under mild salt stress.** Squares represent wild type **(A)**, diamonds the *ggpS* inactivation mutant **(B)**, circles the *glpK* inactivation mutant **(C)**, and triangles the *slr1670* inactivation mutant **(D)**. Filled symbols represent optical density values and correspond to the left y-axes; empty symbols represent extracellular glycerol concentrations c, measured in mmol/L and correspond to the right y-axes. Cells were grown in BG11 medium buffered to an initial pH of 8.0 with 10 mM TES/KOH and supplemented with 200 mM NaCl. Error bars represent the standard deviation of at least two biological replicates. Error bars that are not visible are smaller than the respective data point symbol.

**FIGURE 5:**
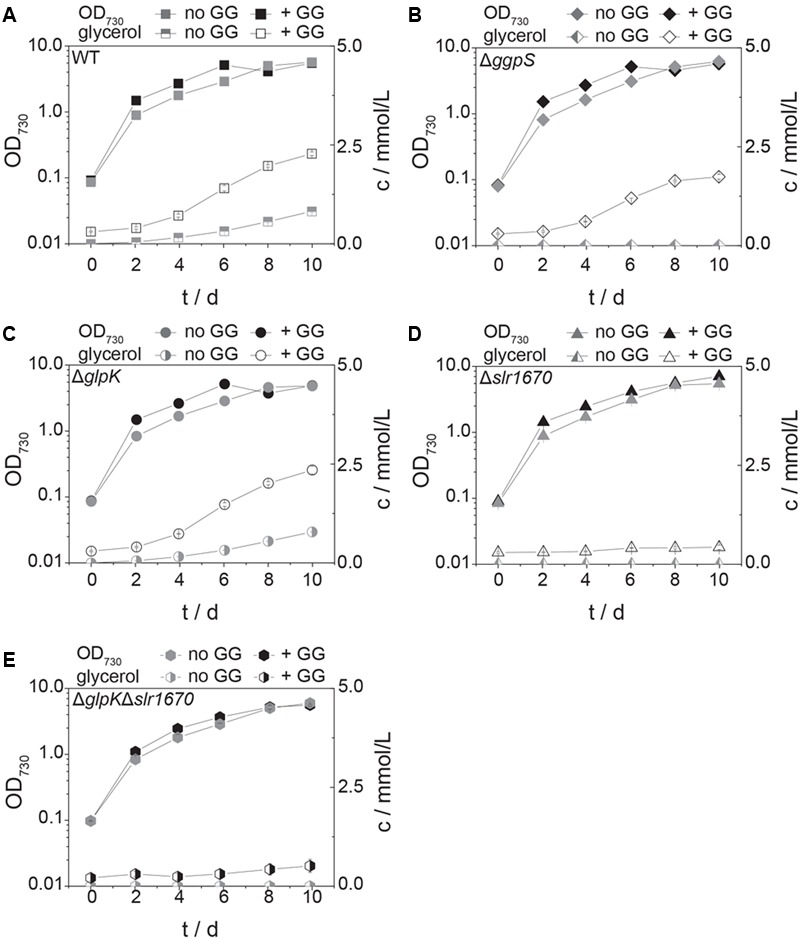
**Glucosylglycerol degradation is abolished in the *slr1670* inactivation mutant.** The *ggpS* disruption strain cannot synthesize GG, but does produce glycerol in the presence of extracellular GG. Squares represent the wild type strain **(A)**, diamonds the *ggpS* inactivation mutant **(B)**, circles the *glpK* inactivation mutant **(C)**, triangles the *slr1670* inactivation mutant **(D)**, and hexagons the *slr1670/glpK* double inactivation mutant **(E)**. Filled symbols represent optical density values and correspond to the left y-axes; half-filled or empty symbols represent glycerol concentrations c, measured in mmol/L and correspond to the right y-axes. Cells were grown in BG11 medium buffered to an initial pH of 8.0 with 10 mM TES/KOH, and supplemented with 200 mM NaCl in the presence and absence of 10 mM GG. Error bars represent the standard deviation of at least two biological replicates. Error bars that are not visible are smaller than the respective data point symbol.

When grown in presence of 200 mM NaCl, wild type *Synechocystis* accumulates small amounts of glycerol in the extracellular medium (Savakis et al., 2015 and **Figure 4A**) The transcript of *glpK*, encoding glycerol kinase, was reported to be upregulated under salt stress conditions (Billis et al., 2014). We expected that in the absence of other assimilation reactions, a strain deficient in *glpK* would produce an increased amount of glycerol when facing salt stress. Interestingly, instead, glycerol production remained unaltered in a strain in which *glpK* was disrupted with a chloramphenicol resistance cassette (**Figures 4A,C** and **5A,C**). This finding suggests that *glpK* might not be involved in the assimilation of glycerol under the conditions tested.

To test whether instead the appearance of glycerol in the extracellular medium is dependent on the presence of intracellular GG, we analyzed a mutant strain in which *ggpS* is disrupted via insertion of a kanamycin resistance cassette (Du et al., 2013). This strain is sensitive to moderately high salt concentrations (∼400 mM NaCl, Supplementary Figure S1), as observed previously (20). Growth at lower concentrations of NaCl (i.e., 200 mM), however, was unaffected (Supplementary Figure S1F). In the supernatant of liquid cultures of this strain, no glycerol could be detected upon the addition of salt (**Figure 4B**). This indicated that GG is a precursor of glycerol under these conditions.

Significantly, within the genomic context of *ggpS* and *glpK* there is also a gene, whose transcript was shown to be upregulated under salt stress conditions (Dickson et al., 2012; Qiao et al., 2013; Billis et al., 2014). The open reading frame upstream of *glpK, slr1670*, has a translated length of 885 amino acids. The Slr1670 protein belongs to the GHL5 family of hypothetical glucoside hydrolases (Naumov and Stepushchenko, 2011). In order to investigate the role of Slr1670, a mutant strain was constructed in which this ORF was disrupted by a kanamycin resistance cassette (**Figure 1B**). Growth of the Slr1670 disruption strain was unaffected by salt (Supplementary Figures S1A,D,G,J). However, no glycerol was detectable in the extracellular medium when this strain was grown in BG11 medium supplemented with 200 mM NaCl (**Figure 4D**).

We then added GG to cells of the Δ*slr1670* strain, and analyzed the extracellular glycerol concentration in relation to the amount of glycerol formed in related deletion mutants (**Figure 5**). In the wild type strain, the concentration of extracellular glycerol was increased as compared to the control condition (no additional GG, **Figure 5A**). The *ggpS* disruption strain accumulated extracellular glycerol only when GG was added (**Figure 5B**). The amount of glycerol was lower than in the wild type strain. When GG was supplied to the Δ*glpK* strain, the extracellular accumulation of glycerol was increased and comparable to that of the wild type (**Figure 5C**). In all strains tested, the addition of extracellular GG led to an increase in growth rate in the exponential phase (**Figures 5A–D**). When GG was added exogenously to the *slr1670* disruption mutant, no glycerol was formed (**Figure 5D**). These findings are indicative of Slr1670 playing a role in glycerol production from GG.

**FIGURE 6:**
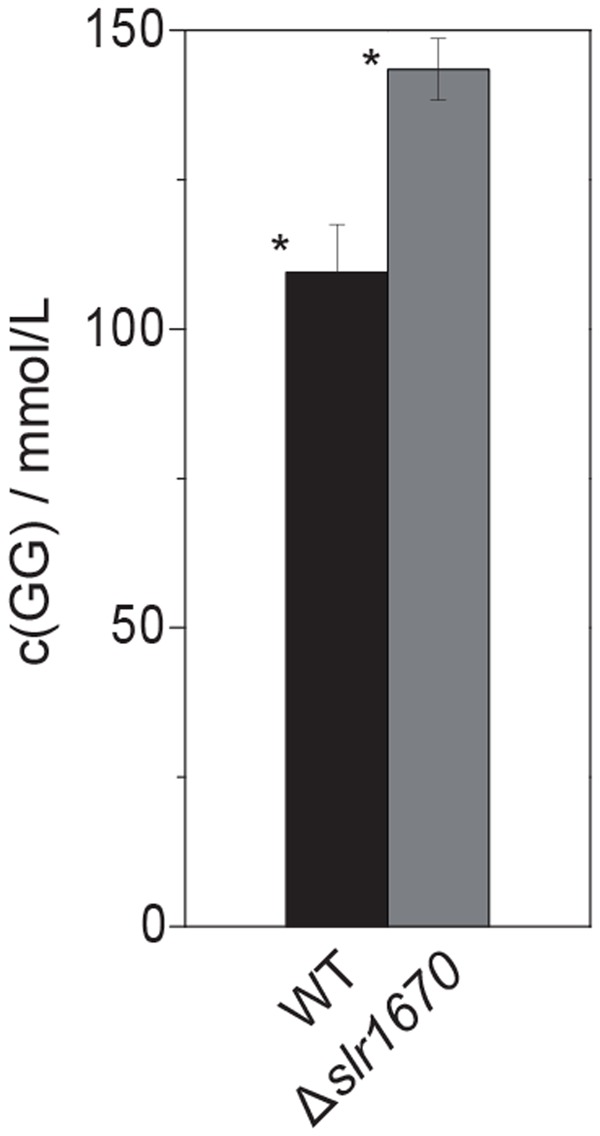
**Inactivation of *slr1670* leads to increased accumulation of intracellular glucosylglycerol.** Cells were grown in BG11, supplemented with 200 mM NaCl and 10 mM TES buffer, at an initial pH of 8.0 to OD_730_ values of 6–7. For each strain, three biological replicates were analyzed. For the extraction, three technical replicates were taken from every biological replicate. Intracellular concentrations were calculated assuming a culture with an OD_730_ of 1 contains 0.2 g of dry weight per liter and assuming that 1 mg dry weight corresponds to 1 μL intracellular volume. Error bars show standard deviations calculated over all data points for a given strain (9). The asterisk denotes statistical significance (*P* < 0.001).

To make sure that the absence of glycerol formation could not be attributed to polar effects of the *slr1670* disruption [e.g., (over-) expression of *glpK* from the promoter of the kanamycin resistance cassette], a mutant deficient in both *slr1670* and *glpK* was constructed. Under salt stress, this strain failed to accumulate glycerol in the extracellular medium (**Figure 5E**). The *slr1670* deletion strain showed increased intracellular concentrations of GG (**Figure 6**), corroborating the hypothesis that Slr1670 is involved in GG degradation. Taken together, these findings suggest that Slr1670 is required for the degradation of GG to glycerol.

We used genome-scale modeling, to analyze whether a possible growth advantage could be conferred by the ability to degrade GG. We started with the model published by Nogales et al. (2012), and extended it with previously published data and with the new information acquired here. Since inactivation of the ABC transporter involved in GG uptake leads to extracellular accumulation of GG (Hagemann et al., 1997; Mikkat and Hagemann, 2000), a leakage reaction from the cytoplasm to the periplasm was introduced. In this state, the model would not predict synthesis of GG when maximizing growth rate. Therefore, a sink reaction for cytoplasmic GG was introduced (**Table 4**; **Figure 2**). Since it is at this point unknown whether cleavage of GG occurs via hydrolysis or phosphorolysis (as described by Nihira et al., 2014 for the *B. selenitireducens* enzyme), both reactions were included.

Phosphorolysis of GG, similar to the reaction catalyzed by the enzyme of *B. selenitireducens*, yields phosphorylated glucose (Nihira et al., 2014). We therefore expected a clear preference for this reaction over the hydrolysis reaction. Accordingly, we simulated growth for phosphorolytic cleavage for a range of GG uptake fluxes, qGG, and divided the obtained rates by those obtained for hydrolytic cleavage (Supplementary Figure S2A). At low uptake rates (low qGG values), no difference in growth rate was predicted for either phosphorolysis or hydrolysis. Only at very high uptake rates was the phosphorolysis reaction beneficial, but even there, the predicted increase in growth rate was minor (<2%). Interestingly, the genome-scale model makes similar predictions for the benefit of glycerol utilization: for low values of qGG, no benefit for the assimilation of glycerol is predicted (Supplementary Figure S2B). Even at very high values, the increase in ratio is very small (<2%).

To estimate a physiological range for qGG, we used the glycerol production data from the strains supplemented with extracellular GG (**Figure 5**). The Δ*ggpS* mutant is unable to synthesize GG, and extracellular glycerol consequently must stem from the degradation of exogenous GG exclusively (**Figure 5B**). We therefore used the glycerol production values from this strain to constrain qGG in the genome-scale model. Next, we simulated photo(hetero)trophic growth (μ) at 30 μE/m^2^/s with the resulting qGG values for wild type, Δ*ggpS*, Δ*glpK*, and Δ*slr1670*. To estimate if the degradation of GG results in increased fitness for these qGG values, we divided the growth rate of the respective strains by the growth rate of the Δ*slr1670* mutant and compared these values to the experimentally determined data (**Table 5**). In the exponential phase (days 0–4) there is excellent agreement between simulation and experiment. Between day 4 and day 6 and between day 8 and day 10, the predicted increase is lower than in the experiment. As the culture increases in density, the absolute value of photons that a single cell perceives decreases. This results in slower (linear) growth (due to light limitation). Under the assumption that the contribution of GG utilization to growth remains constant, the difference in growth (expressed as the ratio) between a strain that utilizes GG, and one that cannot, will therefore be amplified under low light conditions. Since we used a constant photon uptake rate for the simulations, it is expected that at later time points, the effect of GG utilization is underestimated. In the strains that do have a functional *slr1670*, on day 8, there is a drop in optical density (**Figures 5A–C**), which results in negative values for the experimentally determined growth rates. Since the model can only predict growth, a comparison between the experimental and simulated ratios is not meaningful for this and the adjacent intervals. The cause of this transient drop in OD is at present unknown. A possible explanation is that the degradation of GG, and utilization of the glucose part thereof, leads to a drop in intracellular osmotic pressure. Transient eﬄux of water could then reduce the cell volume and hence, the optical density.

**Table 5:** Growth rates of *Synechocystis* wild type, *ΔggpS* and *ΔglpK* divided by the growth rate of *Δslr1670*.

		μ/μ [%]					
Interval [days]	qGG [mmol ⋅gDW^-1^ h^-1^]	WTΔslr1670		ΔggpSΔslr1670		ΔglpKΔslr1670	
		sim.^i^	exp.^ii^	sim.^i^	exp.^ii^	sim.^i^	exp.^ii^
0–2	0.008	103.9	100.9 ± 2.4	103.9	105.7 ± 2.3	103.9	102.8 ± 3.5
2–4	0.015	107.4	109.4 ± 11.0	107.4	106.1 ± 12.2	107.4	104.6 ± 10.3
4–6	0.014	106.9	122.8 ± 5.6	106.9	123.3 ± 5.4	106.9	128.7 ± 11.2
6–8	0.009	104.6	-73.7 ± 36.8	104.6	-42.8 ± 24.6	104.6	-106.9 ± 21.7
8–10	0.003	101.5	135.7 ± 41.8	101.5	93.0 ± 31.6	101.5	120.9 ± 59.8

*qGG values were calculated using glycerol production data of the *ΔggpS* mutant (**Figure 5**).*

*^i^ sim.: simulated; at physiological uptake fluxes of GG, the model does not predict a difference for the mode of cleavage (phosphorolytic versus hydrolytic, Supplementary Figure S2A). Similarly, utilization of the glycerol part does not lead to increased growth rate (Supplementary Figure S2B). ^ii^ exp.: experimental; error margins represent standard deviations of three replicates.*

Until now, Slr1670 is annotated as a hypothetical protein, so we decided to study its phylogenetic distribution. A PSI BLAST of the translated sequence of Slr1670 yielded around 90 homologs, which are distributed among the cyanobacteria, α-proteobacteria and Archaea (see **Figure 3** for a condensed tree, including only the cyanobacterial species and Supplementary Figure S3 for the full phylogenetic tree). In many of the cyanobacterial strains, also homologs of *ggpS, ggpP*, and *glpK* could be identified (**Figure 3**). Notably, a homolog was also found in *M. chthonoplastes*, a strain that has been shown to ferment GG under dark conditions, thereby utilizing the glucose moiety, but excreting glycerol (Moezelaar et al., 1996).

## Discussion

Production of one GG molecule requires fixation of 9 CO_2_ molecules. From a cellular/physiological point of view, GG is an energetically costly compound with often transient use only. It is therefore conceivable that systems have evolved to reduce energy losses related to the synthesis of this osmolyte. The GgtABCD system takes up GG that is lost from the cells due to leakage from the cellular cytoplasm; inactivation of this system leads to pronounced extracellular accumulation of GG (Hagemann et al., 1997; Mikkat and Hagemann, 2000). Upon transition from a high-salt to a low-salt environment, osmoprotective compounds are no longer needed to maintain turgor pressure, so cells able to salvage the nine carbon atoms of GG are at an advantage. In line with this, the adjusted genome scale model of *Synechocystis* predicted an increase in growth rate when extracellular GG was made available (**Table 5**).

Marine cyanobacteria may ferment a portion of their osmoprotectants (Stal and Moezelaar, 1997). For example, *M. chthonoplastes* ferments GG, using only the glucose moiety and excreting the glycerol (Moezelaar et al., 1996). In *M. chthonoplastes*, GG was used as a substrate for fermentation during the night. Interestingly, a gene homologous to *slr1670* was also found in the genome of *M. chthonoplastes* (**Figure 3**), suggesting that the corresponding protein is involved in the degradation of GG in this strain.

At this moment, it is not known if the degradation of GG, observed under continuous illumination in *Synechocystis*, a freshwater organism, serves a regulatory purpose or is the result of a constitutive reaction that becomes more meaningful in the dark. During growth with constant incident illumination, the average amount of light per cell decreases. The individual cells, however, perceive something different. At a given moment, the cells located close to the light source receive light at a high intensity, while a cell in the center of the culture receives fewer photons or none at all. Increases in culture density therefore may lead to increased periods of darkness from the perspective of an individual cell. This is corroborated by the observation that more glycerol is excreted per cell in the late versus the early phase of the experiment (**Figures 4** and **5**). This notion might explain why glucose recycling becomes active during the day.

Cellular release of glycerol by both *Synechocystis* and *M. chthonoplastes* is in agreement with the prediction from the genome scale model, i.e., that glycerol utilization does not lead to increased growth at physiological levels of the GG assimilation reaction (Supplementary Figure S2B). The mutant strains deficient in degradation of GG are valuable tools to study the kinetics of GG synthesis.

Under industrial production settings, *Synechocystis* may be exposed to variations in osmotic stress. It is important to be able to simulate the behavior of this organism under such condition in order to facilitate its usage in the direct conversion of CO_2_ to products of interest in a sustainable fashion (dos Santos et al., 2014). The addition in the model of the ability to synthesize, transport and degrade GG is a necessary step toward the accurate simulation of the growth of *Synechocystis* in such conditions. Additionally, disruption of *slr1670* allows the construction of strains with increased levels of GG, an interesting commodity chemical (Tan et al., 2015).

## Conclusion

We have demonstrated a function for a previously non-annotated protein that has turned out to be required for the re-assimilation of GG. The protein shows homology to glucoside hydrolases and is found in a wide range of cyanobacteria, where its presence correlates with the presence of genes required for GG synthesis. Furthermore, homologs of this protein are also found in the α-proteobacteria and in the domain of the Archaea (Supplementary Figure S3). Strikingly, halotolerant organisms are overrepresented in these two classes of organisms, particularly among the Archaea. Often, homologs obtained had previously been annotated as alpha-amylases. These annotations, however, are purely based on homology and are not supported by experimental evidence. It is also of interest to note that the genomes of some cyanobacterial species encode multiple reading frames that exhibit sequence similarity with Slr1670.

Genome-scale modeling revealed that the increase in growth rate caused by the utilization of the glucose part of GG outweighs the use of the glycerol part by far. This is in line with observations in *M. chthonoplastes*, in which only the glucose part of GG is utilized by the cells. Studies with the purified enzyme will help to elucidate the kinetic properties of Slr1670 and shed light on the mechanism of cleavage of GG.

## Author Contributions

PS and XT conceived the study, designed and carried out the experimental plan, and wrote the paper. CQ and KS implemented the experimental plan. XL, KH, and FBS conceived and supervised the study and wrote the paper.

## Conflict of Interest Statement

KH is advisor to Photanol B.V. The remaining authors declare that the research was conducted in the absence of any commercial or financial relationships that could be construed as a potential conflict of interest.
