# Supplementary material for: Slr1670 from Synechocystis sp. PCC 6803 Is Required for the Re-assimilation of the Osmolyte Glucosylglycerol
Source: Front Microbiol. 2016 Aug 29;7:1350. doi: 10.3389/fmicb.2016.01350 (PMC5002435; doi:10.3389/fmicb.2016.01350)

Supplementary Material

Slr1670 from *Synechocystis* sp. PCC 6803 is required for the re-assimilation of the osmolyte glucosylglycerol

Philipp Savakis, Xiaoming Tan, Cuncun Qiao, Kuo Song, Xuefeng Lu, Klaas J. Hellingwerf, Filipe Branco dos Santos*

*** Correspondence:** Filipe Branco dos Santos: f.brancodossantos@uva.nl

Content:

Figure S1, Figure S2 and Figure S3

Figure S1: Growth of *Synechocystis* mutants is compared in BG11 medium supplemented with 10 mM TES/KOH to an initial pH of 8.0 and with varying amounts of NaCl. The experiments were carried out in 96-well plates. OD values are shown as the difference of the OD and the OD of wells with the same condition but without cells. In some cases, this can lead to ODs < 0. Error bars represent standard deviations of 8 biological replicates.

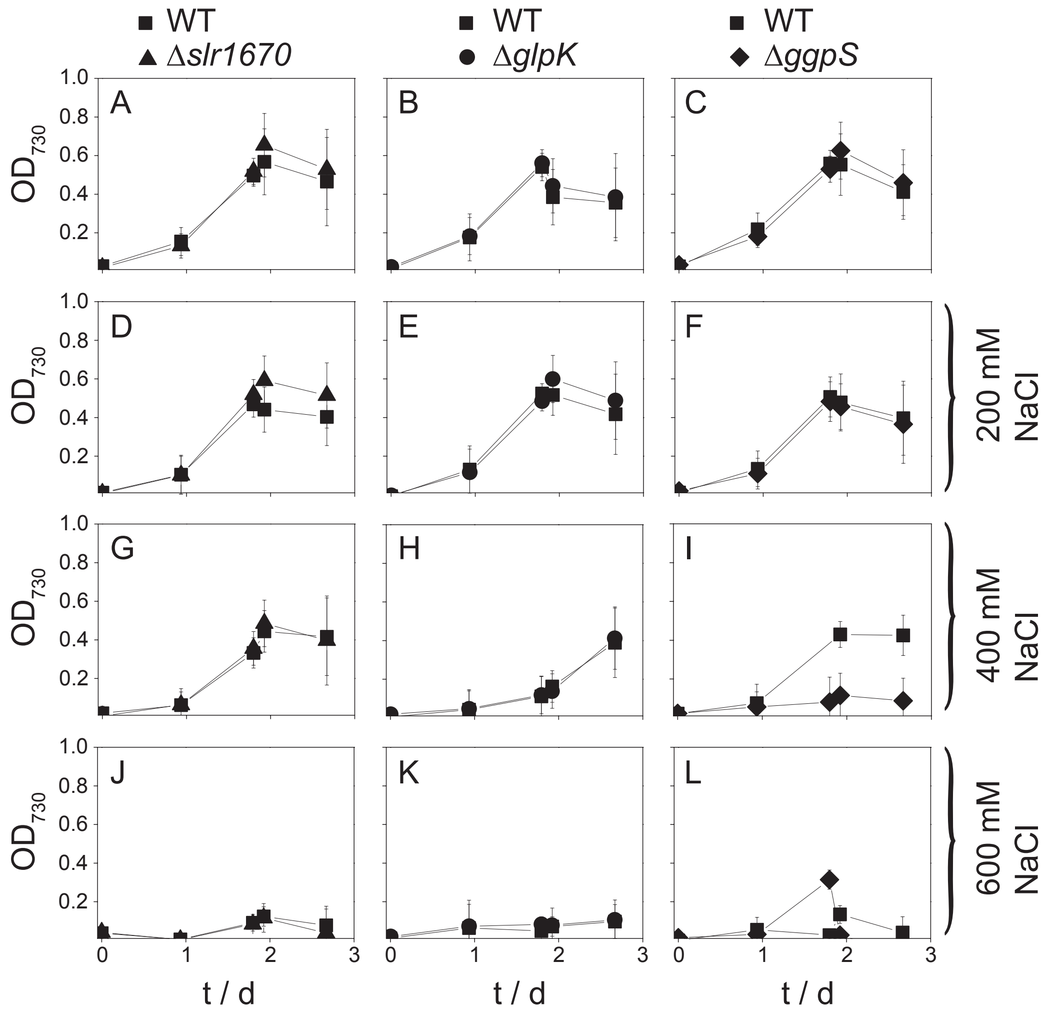

Figure S2: Ratios of simulated growth rates in response to varied GG uptake fluxes. (A): Comparison between phosphorolytic and hydrolytic cleavage of GG. At physiological GG uptake rates, there is no difference between phosphorolysis and hydrolysis. At very high, non-physiological, uptake rates, phosphorolysis of GG leads to higher growth rates than hydrolysis. This effect is slightly larger, when glycerol is not used. (B): Influence of glycerol utilisation. At physiological uptake rates, utilisation of glycerol does not increase growth rate. At very high uptake rates, a minor effect is predicted.

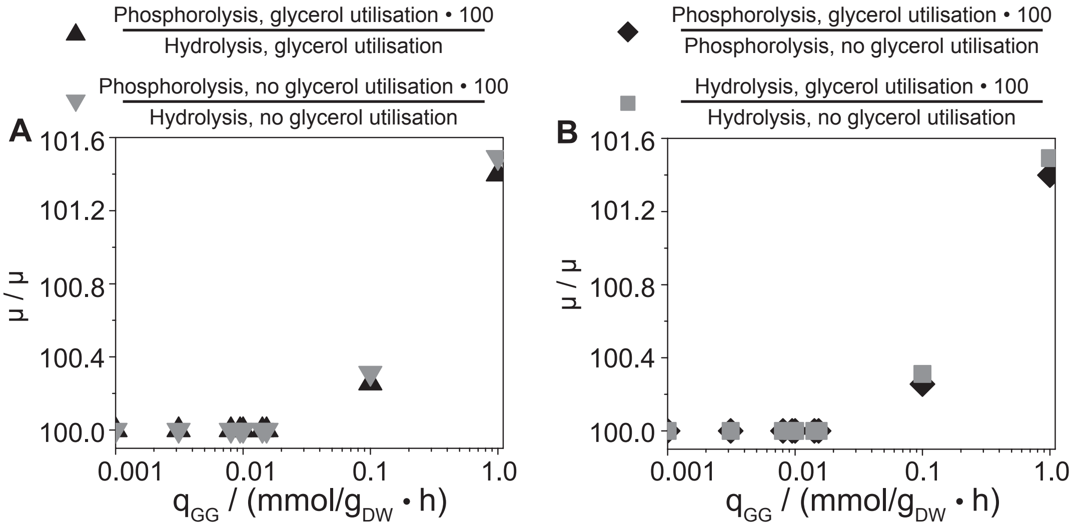

Figure S3: Phylogenetic distribution of reading frames showing sequence similarity to Slr1670. (A) Full tree; for the sake of clarity, large clades are shown collapsed. The corresponding subtrees are shown below. (B) Cyanobacterial subtree. The overwhelming majority of entries belong to the class Oscillatoriophycideae. *Stanieria*, *Myxosarcina* and *Pleurocapsa* are the only representatives of another class, Pleurocapsales. (C) Purple bacteria subtree. All species in this subtree belong to the class α-proteobacteria. With the exception of *Salinarimonas rosea* (Rhizobiales) and *Thalassobaculum salexigens* (Rhodospirillales), all entries belong to the order Rhodobacterales. (D) Archaea subtree. All species in this subtree belong to the class Halobacteria. All species belong to the order Haloferacales, with the exception of *Halapricum* and *Haloarcula*, which belong to the Halobacteriales. Bootstrap values (500 repetitions) are shown on the nodes. Values ≤ 80 are not shown.

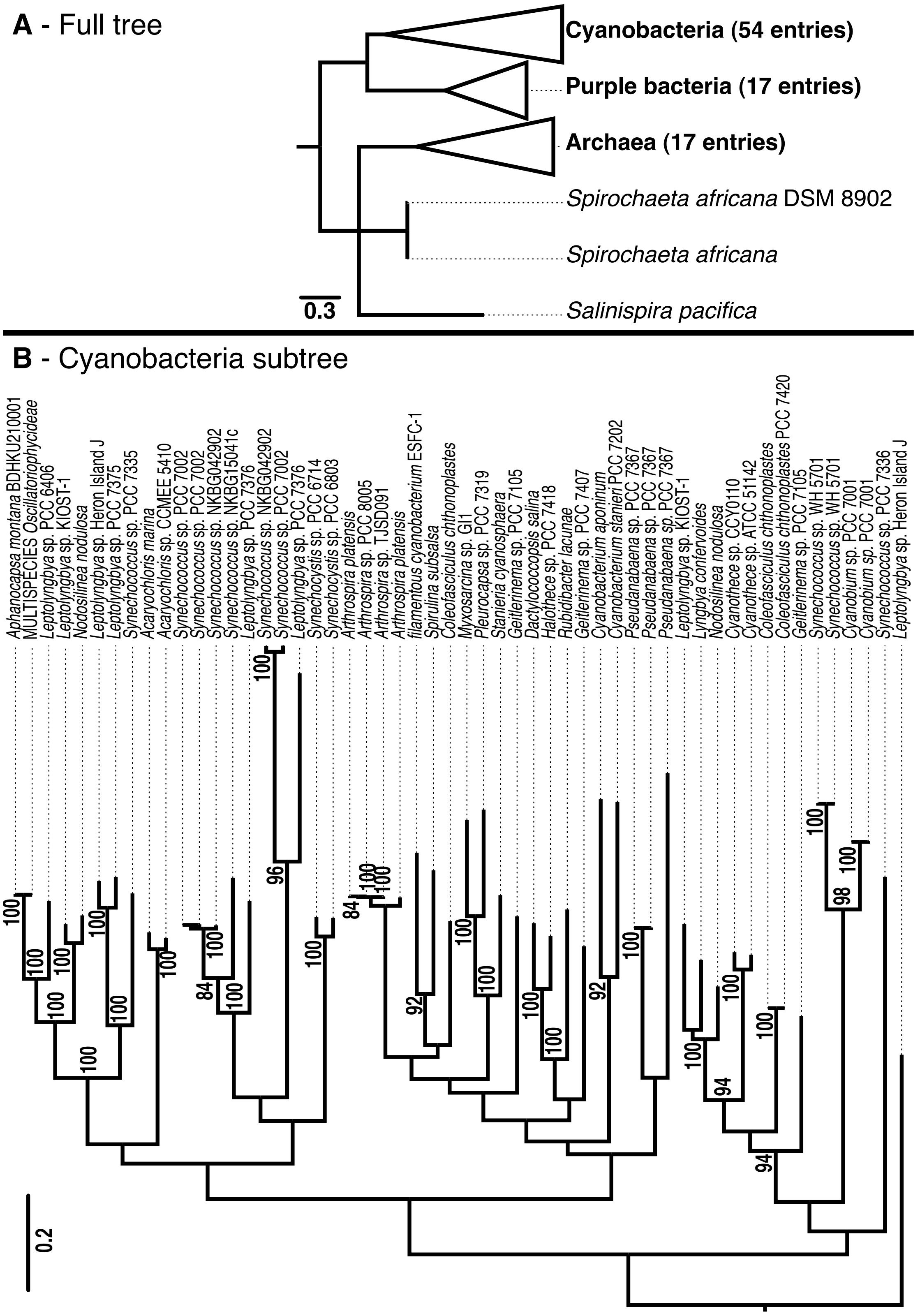
A - Full tree
Cyanobacteria (54 entries)
Purple bacteria (17 entries)
Archaea (17 entries)
Spirochaeta africana DSM 8902
Spirochaeta africana
Salinispira pacifica
0.3
B - Cyanobacteria subtree
Aphanocapsa montana BDHKU210001
MULTISPECIES Oscillatoriophycideae
Leptolyngbya sp. PCC 6406
Leptolyngbya sp. KIOST-1
Nodosilinea nodulosa
Leptolyngbya sp. Heron Island J
Leptolyngbya sp. PCC 7375
Synechococcus sp. PCC 7335
Acaryochloris marina
Acaryochloris sp. CCMEE 5410
Synechococcus sp. PCC 7002
Synechococcus sp. NKBG042902
Synechococcus sp. NKBG15041c
Leptolyngbya sp. PCC 7376
Synechococcus sp. NKBG042902
Synechococcus sp. PCC 7002
Leptolyngbya sp. PCC 7376
Synechocystis sp. PCC 6714
Synechocystis sp. PCC 6803
Arthrospira platensis
Arthrospira sp. PCC 8005
Arthrospira sp. TJSD091
Arthrospira platensis
filamentous cyanobacterium ESFC-1
Spirulina subsalsa
Coleofasciculus chthonoplastes
Myxosarcina sp. GI1
Pleurocapsa sp. PCC 7319
Stanieria cyanosphaera
Geitlerinema sp. PCC 7105
Dactylococcopsis salina
Halothece sp. PCC 7418
Rubidibacter lacunae
Geitlerinema sp. PCC 7407
Cyanobacterium aponinum
Cyanobacterium stanieri PCC 7202
Pseudanabaena sp. PCC 7367
Pseudanabaena sp. PCC 7367
Pseudanabaena sp. PCC 7367
Leptolyngbya sp. KIOST-1
Lyngbya confervoides
Nodosilinea nodulosa
Cyanothece sp. CCY0110
Cyanothece sp. ATCC 51142
Coleofasciculus chthonoplastes
Coleofasciculus chthonoplastes PCC 7420
Geitlerinema sp. PCC 7105
Synechococcus sp. WH 5701
Synechococcus sp. WH 5701
Cyanobium sp. PCC 7001
Cyanobium sp. PCC 7001
Synechococcus sp. PCC 7336
Leptolyngbya sp. Heron Island J
0.2

C - Purple bacteria subtree

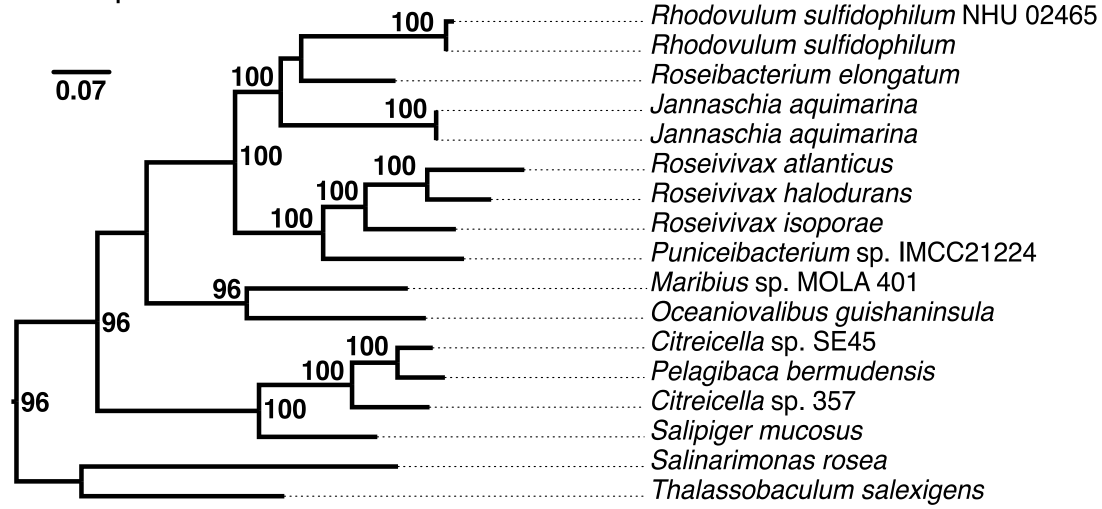

D - Archaea subtree

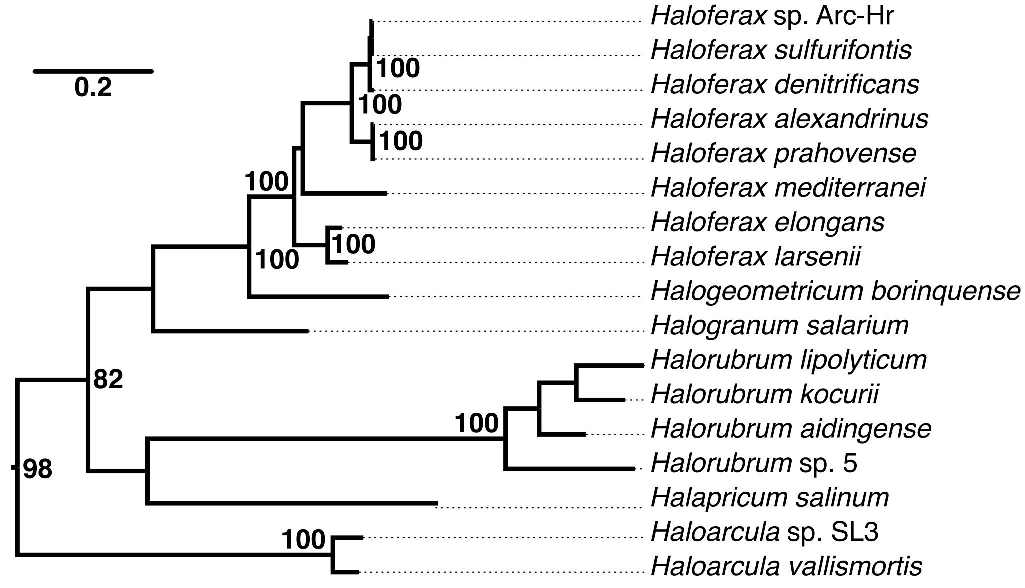

Supplement: Supplementary file 1 [file Image_1.PDF]
